# Pediatric bipolar disorder versus developmental trauma and holistic assessment and care: the contribution of Dr. Ed Levin

**DOI:** 10.3389/fpsyt.2025.1541905

**Published:** 2025-07-07

**Authors:** Peter I. Parry

**Affiliations:** ^1^ Children’s Health Research Clinical Unit, Faculty of Medicine, The University of Queensland, Brisbane, QLD, Australia; ^2^ Department of Psychiatry, College of Medicine and Public Health, Flinders University, Adelaide, SA, Australia

**Keywords:** psychiatric diagnosis, developmental trauma, complex PTSD, overmedicalization, iatrogenic drug use, trauma informed care, pediatric bipolar disorder, childhood maltreatment

## Abstract

This perspective article presents the work of Dr. Edmund (Ed) C Levin (1931-2022), child and adolescent psychiatrist in Berkeley, California. Levin drew from over half a century of continuity of clinical practice with his patients and knowledge of developmental psychopathology. He was witness to a paradigm shift in American psychiatry from what Eisenberg termed a ‘brainless’ to a ‘mindless’ approach in research and clinical practice. He was motivated by concern for medical ethical treatment guided by awareness of the patient’s individual biopsychosocial contributing factors to their predicament and symptoms. He addressed the pediatric bipolar disorder era by championing a recognition of the long-term effects of childhood maltreatment and developmental trauma across the lifespan. His work in both child and youth residential and geriatric residential units exemplified this.

## 1 Introduction

In 1994 Robert Hirschfeld published a memorial paper about the late Gerald Klerman (1928 – 1992), describing Klerman’s contribution to diagnosis in psychiatry: that Klerman “understood and appreciated the importance of descriptive, biological, psychoanalytic, social, interpersonal, and behavioral approaches and was uniquely able to integrate them cogently” (1). In this paper I seek to draw attention to the long career of Dr. Edmund C Levin (1931 – 2022), child and adolescent psychiatrist, who upheld the same principles as Klerman, and was a strong critic of the “Pediatric Bipolar Disorder” (PBD) diagnostic construct. In critiquing PBD, Levin pointed to the importance of understanding the child or young person through a developmental psychopathology lens that included attachment and trauma history.

Levin’s work warrants greater recognition, in particular his clinical oversight of two residential child and adolescent units where the application of trauma-informed care led to much clinical improvement with significant deprescribing and removal, in many cases, of medicalized DSM diagnostic labels.

Dr. Ed Levin stressed the importance of understanding family histories. His father was an immigrant from Lithuania who arrived in New York aged 12 as an unaccompanied minor, made his way to Charleston, West Virginia, peddling eyeglasses from a backpack to miners in the hills. Ed was born March 28, 1931, as youngest of four, eldest brother Charles, Ed’s middle name, having died in infancy. His father served in World War I and died by suicide some years later when Ed was aged 8 years, indelibly imprinting upon him the post-traumatic effects of war and generating an abiding interest in mental health. His mother took Ed and his two sisters to Beverly Hills when he was aged 13 and he became politicized to left wing politics as friends’ fathers (including the ‘Hollywood 10’) were purged of their careers during the McCarthy era.

Levin’s political activism continued while obtaining a BA from UC Berkeley in 1953. Upon graduation he was drafted as a soldier during the Korean War. He did not go to Korea, being discharged because of his political affiliations. However, pragmatic common humanity rather than dogmatic idealism guided his worldview, and these left-wing perspectives moderated after the Soviet Union invasion of Hungary in 1956. The Cold War and experiences of friends during McCarthyism impressed upon him the importance of free speech, peace and necessity of avoiding nuclear war. In 1985 during a period that we now know civilization came within a hair’s breadth of annihilation (2), Levin was the founder and longtime chair of the Committee on the Clinical and Developmental Aspects of the Nuclear Threat in the American Academy of Child & Adolescent Psychiatry (AACAP) and had a letter to *JAMA* published (3) on the issue of adolescent mental health and role of child psychiatrists regarding the effects of this ongoing threat.

Following discharge from the army he worked in a factory in the East Bay area and then as a biostatistician for the California State Department of Public Health. He entered medical school on the GI bill and earned his MD from UCLA in 1962, then returned to the San Francisco Bay area to train in psychiatry and complete his fellowship in child and adolescent psychiatry at Langley Porter Psychiatric Institute, UCSF, by 1968. From that year he started a private practice in Berkeley from where he provided continuity of child and adolescent psychiatric care for over half a century, including for children of former patients. This continuity he said, helped him understand the broad developmental context within which psychopathological and trauma-reactive symptoms manifest. It informed his critique of the PBD diagnostic construct, and other de-contextualized diagnosing and over-medicating management practices.

Levin was on staff of the Alta Bates Summit Medical Center, Berkeley and on its continuing medical education (CME) committee from 1975, serving as chair from 1998 until December 2021. He was active within the Northern California Regional Organization of Child and Adolescent Psychiatry (NCROCAP), serving as president, treasurer or secretary and serving as national assembly delegate from 2013 to 2018. In 2008 NCROCAP awarded him a Lifetime Achievement Award “for outstanding service to his profession, mentoring of young psychiatrists, his writing, and his dedication to and skill with his patients”. He was a Distinguished Life Fellow in AACAP and a Distinguished Life Fellow in the American Psychiatric Association (APA). At the time of his death, he had an academic affiliation with UCSF and was supervising fellows in child psychiatry training.

Levin was first and foremost a clinician, his academic focus was mostly on teaching and conveying to the profession his views that the full context of a child or youth’s life, including family dynamic, psychodynamic and traumatic adverse childhood experiences, must be considered for accurate diagnosing and appropriate management. His curriculum vitae cited a selected 51 conference and grand round presentations mostly on this theme and listed 23 publications, seven in peer reviewed journals, the majority in *AACAP News* as he was advocating to his specialty colleagues.

Dr. Levin was by nature indefatigable. He played weekly social (but highly competitive) basketball into his 80s, and although of short stature as well as great age, he kept pace with teammates and opponents decades younger. I discovered this when over three hours of seven games, he kept effectively blocking my passes and shots for the hoop, on a visit during the APA annual meeting in 2009.

He had a quick wit, engaging warm humor and sublime sense of irony. He embodied a strong sense of justice and the ideals of medical ethics – to provide beneficence and to do no harm to his patients, to respect the autonomy and hear the voice of patients from disadvantaged and traumatized backgrounds. He poured that energy and passion into his work and advocacy and these qualities shone through in his success in the youth residential units as described below. At the age of 91 Dr. Ed Levin was still writing, researching, presenting, supervising and practicing clinically until a few weeks before his passing. He is survived by many friends and colleagues, his wife, Robin Deutsch, three daughters, two grandchildren and four great grandchildren.

## 2 Dr. Levin’s significant contributions

### 2.1 Leadership in confronting ‘Study 329’ findings

During his time as delegate to the AACAP national assembly, he and colleagues lobbied for AACAP to reduce its reliance on pharmaceutical industry funding. They unsuccessfully lobbied to have the AACAP executive acknowledge errors within the publication of Keller et al., 2001 in the *Journal of the American Academy of Child & Adolescent Psychiatry (JAACAP)* (4). Keller et al. was the first publication of GlaxoSmithKline (GSK, formerly SmithKline Beecham – SKB)’s “Study 329” – a randomized controlled trial of paroxetine versus imipramine versus placebo in a cohort of depressed adolescents. The paper in *JAACAP* still concludes “Paroxetine is generally well tolerated and effective for major depression in adolescents”.

However, following criminal prosecution of GSK for among other violations, data fraud that involved Study 329 (5, 6) the Keller et al., 2001 paper became a focus of controversy. Levin and colleagues in the NCROCAP and Central CROCAP, passed motions calling for retraction of Keller et al., 2001 from *JAACAP*. They then brought this motion to the national AACAP Assembly Meeting on 21 October 2014. Levin described (email communication Levin-Parry, 3 November 2014) how the motion to retract or correct Keller et al. was closely defeated at the end of a long day, when many members had left, after filibustering by AACAP executives. A second motion to refer the Study 329 controversy and Keller et al. paper to the AACAP Ethics Committee was deferred but never returned to.

Despite the lack of retraction or correction in *JAACAP*, an independent group of researchers were granted access to GSK’s raw data on Study 329 and published their findings in the *BMJ* in 2015 (7). The title was “Restoring Study 329: efficacy and harms of paroxetine and imipramine in treatment of major depression in adolescence”. This second peer-reviewed publication of the same data found:

“The efficacy of paroxetine and imipramine was not statistically or clinically significantly different from placebo for any prespecified primary or secondary efficacy outcome…. There were clinically significant increases in harms, including suicidal ideation and behavior and other serious adverse events in the paroxetine group and cardiovascular problems in the imipramine group.” [7, p. 1]

### 2.2 Addressing the pediatric bipolar disorder epidemic

I was introduced to Dr. Ed Levin by our mutual friend Prof David Healy, a co-author of the “Restoring Study 329” paper, an international authority on the history of psychiatry and Pharma-Medicine conflicts of interest. Healy had published a critique of PBD (8) and felt that Levin and I could collaborate on the overdiagnosis of bipolar disorder in childhood and resulting iatrogenic harms befalling children through overmedication and neglect of underlying issues such as developmental trauma.

Levin coordinated a symposium for the 2009 APA 162^nd^ annual meeting in San Francisco, involving us plus Prof Glen Elliott (Stanford) and A/Prof Mary Burke (UCSF), titled *Pediatric Bipolar Disorder: A critical look at an American phenomenon* (9). The abstracts for our presentations are in the APA program book in appendix A8 of my doctoral thesis, *‘Paediatric Bipolar Disorder’: Why did it occur, the iatrogenic consequences, and the implications for medical ethics and psychiatric nosology* (10).

The PBD diagnostic criteria deviated from classical descriptions of bipolar disorder and were minted at a few prominent US child psychiatric academic centers in the early 1990s. By 2009 the PBD diagnosis had spread widely within the USA and to a few international, mainly Latin American and Mediterranean child psychiatry centers. Hundreds of papers had been published giving credence to the PBD diagnostic constructs of either ultrarapid cycling (narrow phenotype) or chronic irritability (broad phenotype) (11) PBD.

There were numerous presentations regarding PBD at APA or AACAP conferences, with very little in the way of opposing viewpoints to that time. The dominant narrative in the US was that PBD is real and relatively common. Hundreds of peer-reviewed papers in prominent American psychiatry and pediatric journals, including treatment guidelines authored by leading academics, attested to that. There was pushback against dissenting viewpoints, and to our knowledge our 2009 APA symposium was the first PBD skeptical symposium to be accepted onto the academic program of either an APA or AACAP meeting (9). Dr. Levin’s professional application to the APA program committee achieved this.

The large auditorium was full and appeared split roughly 50:50 into proponents and skeptics of PBD and question time was intense. It was remarked by several attendees that child psychiatrists in academic teaching centers were more likely to be proponents, whereas those with a family oriented clinical community practice were more skeptical. Dr Levin illustrated how changing the treatment culture in a residential agency for youth could lead to far less medicating and better outcomes, as well as removal of PBD diagnosis labels. I presented results of an Australian & New Zealand survey of child psychiatrists that showed only 3% of ANZ child psychiatrists agreed with the PBD diagnosis construct. Prof Glen Elliott critiqued the PBD literature and Dr. Mary Burke addressed bioethical aspects of medicalizing early childhood behavior problems with a bipolar disorder diagnosis.

### 2.3 Developmental trauma disorder as alternative to overmedicalization

Levin presented his experience working for two years in a residential center for foster children in Oakland, where, by educating the staff in trauma-informed care, for which he was a pioneer – the use of medications was able to be greatly curtailed, children’s acting-out behaviors settled, and seclusions for oppositional and aggressive behavior became almost non-existent.

One girl, whom he called ‘Maya’ for anonymity, was tapered off heavy polypharmacy to nil medication and she soared in pro-social behavior, happier mood, social connections, self-esteem, and vastly increased IQ and academic achievement. She no longer needed surgery for being supposedly “tongue-tied” when the extrapyramidal neurological adverse effects resolved with the removal of antipsychotic medication and her speech became fluent. Freed of sedating demotivating adverse effects she excelled athletically in a mainstream school 15 months after discharge from the residential center, still medication free. Maya’s diagnoses of PBD, ADHD, borderline intellectual functioning and PTSD (she had had perhaps as many as 50 foster placements as well as homelessness in her 12 years of life) were dispensed with as her condition was reformulated as Developmental Trauma Disorder (DTD) (12). Maya’s recovery journey, and Levin’s recounting of the tapering deprescribing program, were featured in a series of articles “Drugging Our Kids” by the Californian newspaper *The Mercury News* (13).

Following the APA symposium Levin wrote up his two-year interventional study, which was remarkable for its positive results data. The paper was titled “The challenges of treating developmental trauma disorder in a residential agency for youth”. The paper described the case of Maya, and the changes among the other children and youth aged 6- to 14-years in the two residential units of the Lincoln Child Center, Oakland, plus the trauma-informed care model he introduced and results of this intervention (12).

Levin drew upon the work of van der Kolk and colleagues’ diagnostic construct of Developmental Trauma Disorder (14) and listed the “core components of complex trauma intervention” as: 1) safety; 2) self-regulation; 3) self-reflective information processing; 4) traumatic experiences integration; 5) relational engagement; 6) positive affect enhancement [12, p. 525]. The staff were enabled to provide such care with coaching in consistent and calming communication with the young residents. As an example, aggressive incidents for the first week of February fell from 14 in 2006 to zero in 2008 [12, p. 536].

Levin was an early pioneer of slow medication tapering, that slow withdrawal of psychotropics is important to minimize adverse withdrawal effects that can mimic or aggravate underlying emotional-behavioral symptoms. Quantity of medication in milligrams per day fell across all psychotropic classes (alpha-adrenergic agonists, antidepressants, antipsychotics, mood stabilizers, stimulants) over the two years, by 100% to zero medication in unit A of the residential center, and by 26% in unit B [12, p. 534]. The difference in the two units was a high retention of staff in unit A and high turnover of staff in unit B. Continuity or otherwise of staff was a relevant factor given the insecure and disorganized attachment developmental histories of the young residents, and this difference indicated staff more than resident factors were governing the need for psychotropic medication.

Levin’s manuscript was returned by *JAACAP* almost immediately and rejected as “not scientific enough” by other mainstream psychiatry journals without sending for peer review, until a psychoanalytic journal, *Journal of the American Academy of Psychoanalysis and Dynamic Psychiatry*, recognized the paper’s merits (12). It is a seminal paper deserving of more citations and prominence. It should have been a key paper to reverse the overmedicalization of childhood emotional and behavioral problems of recent decades, for which PBD and antipsychotic medication regimes were emblematic.

Levin and I continued to collaborate and presented a similar themed symposium in Australia in 2010 at the Royal Australian and New Zealand College of Psychiatrists’ Faculty of Child & Adolescent Psychiatry annual conference. We co-authored two papers addressing the fact that PBD was a controversial diagnostic entity: “Conflict of interest as a possible factor in the rise of pediatric bipolar disorder” in *Adolescent Psychiatry* (15), and “Pediatric bipolar disorder in an era of ‘mindless psychiatry’” in the *Journal of Trauma and Dissociation* (16).

The first of those papers described revelations of conflicts of interest involving PBD researchers from subpoenaed internal industry documents released following criminal litigation of pharmaceutical companies. Levin had always avoided pharmaceutical industry funding, viewing it as a corrupting temptation. These documents confirmed his and my fears. The documents indicated companies wanted an expansion of the diagnostic boundaries of bipolar disorder because patents for antidepressants were expiring whereas patents for second generation antipsychotics (SGAs) were still fresh. The marketing plan was to increase SGA prescriptions by rebranding them as mood stabilizers for an allegedly greatly enlarged pool of bipolar disorder sufferers (10, 17).

The second paper placed PBD in a historical context, using Leon Eisenberg’s terms of “brainless psychiatry” and “mindless psychiatry” (18). We found that in recent decades the converging factors that led to the PBD epidemic had been:

A tendency within psychiatry and society to neglect trauma and attachment insecurity as etiological factors; the ‘atheoretical’ but by default biomedical premise of the DSM since its 1980 third edition; the influence of the pharmaceutical industry in research, continuing medical education, and direct-to-consumer advertising; and inequality in the U.S. health system that favors ‘diagnostic upcoding’. [16, pp. 51-52]

The PBD epidemic remained mostly confined to the USA and was a reification of emotionally dysregulated childhood behavior into a mental disorder (19). Contextual factors that Levin exhorted his profession to consider were neglected. As Levin and I stated, citing Harvard professor of child psychiatry Judith Herman, “society is biased against the acknowledgment of trauma” [16, p. 57], and this bias can infect mental health professionals. The iatrogenic adverse effects upon children and adolescents of this were erroneous diagnostic labelling, of missing real diagnoses and causative environmental factors, and of harms from overmedicating.

In 2018 Levin turned his attention to the push for widespread mental health screening for depression in youth. He published in *AACAP News* an article “Depression screening: pros and cons”, that while this was a noble ideal, the risk of overmedicalization was significant. He followed in 2020 with a peer reviewed paper, “Adolescent depression screening: not so fast” (20), documenting the marketing driven role of the pharmaceutical industry in funding mental health screening tools for adolescents. He noted:

when we use a self-administered set of leading questions and a set of algorithms to diagnose and to determine when to initiate medication; we are encouraging the *deprofessionalization* of medicine and disconnecting ourselves from a fuller relationship with our patients…. If we are prepared to use our listening skills in the course of a respectful, empathic, comprehensive evaluation, we get … a richer understanding of the patient, a more accurate diagnosis, a more workable treatment plan, and better treatment compliance than one can obtain with a depression screening device. [20, pp. 67-68]

In 2013, Levin had convened another symposium at the 166^th^ APA annual meeting, again in San Francisco (21). I presented a systematic review of the PBD and Disruptive Mood Dysregulation Disorder (DMDD) literature that revealed a gross lack of examination for maltreatment, attachment and family dynamic factors. Prof Stuart Kaplan, author of “Your Child Does NOT have Bipolar Disorder” (22) addressed the problems of structured questionnaires and rating scales for diagnosing PBD and critiqued the “Course and Outcome of Bipolar Youth” (COBY) study on this basis, and Dr. Stuart Bair, presented a historical context for the rise of PBD due to paradigm shifts in child psychiatry. Levin’s paper was from his more recent experiences in geriatric psychiatry.

### 2.4 Addressing lifelong effects of developmental trauma

By this stage Levin’s contract at the Oakland child residential center had been terminated ostensibly due to reduction in beds, but as he confided to me, senior management expressed concerns that not enough psychotropic medication was being prescribed. He had subsequently taken a psychiatric consultancy role at a residential aged care facility. Again, money was a driving factor for the organization, but this time psychotropic drug reductions, not increases, benefited the agency’s bottom line. As Levin described in his conference paper:

On hearing that I had been able to reduce the amount of psychotropic medications used in the child program by 80%, she offered me a job for as many hours as I wanted. The major reason for her hiring me involved the fact that the agency got a set fee for each enrollee and all the costs of care came out of that. Problematic for their bottom line, psychiatric medication was a huge budget item.

Levin’s geriatric psychiatry experience, as he described in this conference paper, “Developmental Trauma Disorder is not just for kids: mental and physical consequences of early trauma in a geriatric population”, was that he “found much of what I had learned as a Child Psychiatrist was applicable to geriatric work” (23). Of the 56 geriatric patients Levin evaluated during a 16-month contract, he assessed 37 as not having DTD and 19 as having it. DTD was described as:

The trauma was early, severe, chronic, and typically perpetrated by one or more persons close to the victim. Additionally, each had by history, currently or both, multiple symptomatic features of Developmental Trauma Disorder, such as poor impulse control, poor attachments, little tolerance for affect, difficulty with mood regulation, and frequently, substance abuse.

He noted that of the 37 non-DTD patients, three had chronic PTSD not of a DTD nature, two being Vietnam War veterans, and a fourth had remarkable resiliency despite extremely traumatic childhood experiences of war, atrocities and attachment loss. Citing the work of Dallam (24) regarding lifelong consequences of childhood maltreatment, and Felitti (25) on Adverse Childhood Experiences (ACEs), Levin explained:

Physiologic responses to trauma, which include dysregulation of the hypothalamic-pituitary-adrenal axis, lead to the release of multiple hormones relating to stress *(e.g., cortisol, catecholamines, opioids, neurotransmitters)* which may remain elevated over time and which are difficult to modulate later as feedback mechanisms go awry.

These mental and physical health consequences were evident in the DTD group had presented to aged care an average 5 years chronologically, but not biologically, younger than the non-DTD group. The DTD group was over-represented with African American and female patients, reflective of societal factors for childhood maltreatment.

As with the childhood residential center, staff had stressful jobs challenged by demanding resident behavior. Through discussions with staff Levin noted:

Staff generally proved to be curious about developmental trauma and its effects. They began thinking in more psychodynamic ways. When concepts of transference and counter-transference were introduced, they became appreciative of their own feelings and of those of others in relation to what was terribly difficult and demanding work.

Levin found that major psychiatric disorders (schizophrenia, major depression, bipolar disorder) were more prevalent in the non-DTD group (46%) than in the DTD group (26%). In essence, developmental trauma itself, without psychotic disorder or severe major depression was underlying the premature ageing and morbidity of a group of geriatric patients. Additionally, Levin found diagnosing the DTD enabled tapering of some of the patients’ antipsychotic medications, as the challenging behavior was addressed in a trauma-informed therapeutic context.

His observations of overmedicalized treatment of both young and old patients in the Oakland region impressed on him the subtle effects of race in underestimating and exacerbating trauma. He also noted the effects of systemic racism in the PTSD histories of Black veterans. In 2021, a few months before his death, his most recent paper, “An Exploration of Implicit Racial Bias as a Source of Diagnostic Error” was published in the *American Journal of Psychoanalysis* (26).

## 3 Conclusion

Dr. Ed Levin brought into clinical practice the knowledge that childhood maltreatment and developmental trauma underlie much of the psychopathology that a biomedical reductionist psychiatric paradigm had overmedicalized as PBD and other diagnoses. While convening the academic program at a child psychiatric teaching faculty, he also provided over a half century of long-term psychotherapeutic and holistic psychiatric care to his private patients. This provided him a longitudinal perspective that is lacking in the prevalent modus of checklist diagnosis and prescribing algorithms. He also pioneered slow tapering of psychotropic medication long before its current rise in practice.

Although DTD was not accepted for DSM-IV or DSM-5, the ICD-11 now classifies such symptomatology as Complex PTSD. This recognition by the main international diagnostic manual is a corrective for the misdiagnosis, overmedicating, and lack of proper psychotherapeutic interventions and trauma-informed care that characterized the PBD era.

Dr. Levin’s efforts in both pediatric and geriatric psychiatric residential centers ran counter to an overly biomedical reductionist or, to quote Eisenberg as we had (18) – ‘mindless psychiatry’ treatment paradigm. He leaves a legacy to recognize and address the effects of developmental complex childhood trauma across the whole of the lifespan.

## Data Availability

The original contributions presented in the study are included in the article/supplementary material. Further inquiries can be directed to the corresponding author.
